# Acute Fatty Liver Disease of Pregnancy in the Second Trimester

**DOI:** 10.1155/2020/6705784

**Published:** 2020-06-15

**Authors:** Faraz Afridi, Michael Feely, Raju Reddy

**Affiliations:** ^1^Division of Pulmonary, Critical Care and Sleep Medicine, Department of Medicine, College of Medicine, University of Florida, 1600 SW Archer Road, P.O. Box 100225, Gainesville, FL 32610-0225, USA; ^2^Division of Pathology, Immunology and Laboratory Medicine. Department of Medicine, University of Florida, 1600 SW Archer Road, PO Box 100275, Gainesville, FL 32610-0275, USA

## Abstract

Acute fatty liver of pregnancy (AFLP) is a rare disorder that typically presents in the third trimester. We report a case of a 21-year-old woman with a history of intrauterine fetal demise at 19 weeks' gestation who developed fulminant liver failure 1 week after the fetal demise. She was diagnosed with AFLP as per the Swansea criteria. An orthotopic liver transplant was attempted but was unsuccessful. AFLP usually presents between the 30th to 38th weeks of gestation. However, it can occur in the postpartum period after only 19 weeks of gestation as highlighted in our case.

## 1. Introduction

Acute fatty liver of pregnancy (AFLP) is a rare disorder with an incidence ranging from 1 : 7000 to 1 : 15000 [1]. Most cases occur in the third trimester, and a few occur postpartum [2]. To date, there are only three reported cases of AFLP in the second trimester [3–5]. Ours is a case of a 21-year-old woman diagnosed with AFLP after a spontaneous fetal demise at 19 weeks' gestation. A high index of suspicion may be necessary in atypical cases to make an accurate diagnosis. Timely recognition is vital as appropriate management can reduce mortality from 85% to <10% [1].

## 2. Case Presentation

A 21-year-old woman with a history of intrauterine fetal demise at 19 weeks with resulting spontaneous delivery followed by dilatation and curettage (D&C) presented to the hospital 1 week later with a 2-day history of progressively worsening right upper quadrant abdominal pain and nonbilious, nonbloody emesis. She denied any dysuria, diarrhea, and vaginal discharge. She had no significant past medical history including hypertension during her pregnancy. Her only surgical history consisted of the recent D&C. She denied using tobacco, alcohol, and illicit drugs. She was on ursodeoxycholic acid only prior to admission.

An initial exam was significant for an ill appearing obese woman (body mass index 34 kg/m2) in moderate distress due to pain and normal vitals. Her abdominal examination was significant for the right upper quadrant and epigastric tenderness without distention and rigidity. Gynecologic examination revealed old blood clots but no purulence. The remainder of her exam was normal. Initial labs were significant for aspartate aminotransferase (AST) 49 IU/L (normal 0-37 IU/L), alanine aminotransferase (ALT) 70 IU/L (normal 0-35 IU/L), alkaline phosphatase (ALP) 80 IU/L (33-123 IU/L), total bilirubin 1.3 mg/dL (normal 0-1 mg/dL), and lipase 116 U/L (normal 0-70 u/L). Basic metabolic panel, complete blood count, coagulation parameters, ethanol level, and urine drug screen were within normal limits. Acetaminophen and ethanol levels were undetectable.

Differentials at this point included pelvic infection given the recent D&C, pancreatitis, and cholecystitis. Ultrasound of the abdomen revealed increased echogenicity of the liver consistent with steatosis, gallbladder sludge, and normal common bile duct. Computed tomography (CT) of the abdomen and pelvis was negative for any acute intra-abdominal process. Pelvic ultrasound was unremarkable as well. She was started on vancomycin 1 g q12h and piperacillin-tazobactam 3.375 g q8h. The patient quickly deteriorated on hospital day 2 requiring transfer to the medical intensive care unit for altered mental status requiring intubation. CT of the head was negative for an acute intracranial process. Repeat laboratory work-up revealed sudden rise in her liver function tests (LFTs)—AST increased to 4068 IU/L, ALT increased to 1700 IU/L, total bilirubin increased to 4.8 mg/dL, conjugated bilirubin increased to 2.8 mg/dL (normal 0-0.2 mg/dL), and ALP increased to 98 IU/L—and she developed new leukocytosis (WBC 13.8 × 10^3^/*μ*L) (normal 4–10 × 10^3^/*μ*L). Other significant findings included new onset coagulopathy, hypoglycemia, lactic acidosis, and hyperammonemia at 206 *μ*mol/L (normal 18-72 *μ*mol/L). The patient was started on lactulose.

An extensive work-up for fulminant hepatic failure was pursued—repeat acetaminophen levels, methanol, hepatitis panel, autoimmune work-up including antinuclear antibody (ab), anti-smooth muscle ab, anti-mitochondrial ab, anti-liver kidney microsome ab, anti-soluble-liver-antigen ab, anti-cardiolipin ab, anti-beta-2 glycoprotein ab, and anti-lupus anticoagulant ab, ceruloplasmin levels, viral studies for Cytomegalovirus, Herpes Simplex 1, Herpes Simplex 2, Epstein Barr, and Varicella Zoster, uric acid level, alpha-fetoprotein (AFP) level, and hemochromatosis screen.

Other differentials for the sudden rise in LFTs should also include acute portal vein thrombus and pregnancy-related etiologies such as physiologic changes in pregnancy, hyperemesis gravidarum (present in the 1st trimester), intrahepatic cholestasis of pregnancy (ICP), preeclampsia, HELLP syndrome (hemolysis, elevated liver enzymes, and low platelets), and acute fatty liver of pregnancy (AFLP). While infections and autoimmune processes can present acutely, hemochromatosis is an indolent process and was an unlikely etiology. Tylenol levels and hepatitis panel were negative. ICP was a possible etiology, however, the patient did not report any history of pruritis prior to admission. There is some clinical overlap of HELLP syndrome, preeclampsia, and AFLP which can often make it difficult to differentiate. While all these entities can have marked elevation of LFTs, the patient had no thrombocytopenia or hemolysis thus ruling out HELLP syndrome. The patient also had no evidence of hypertension or proteinuria prior to admission ruling out preeclampsia. Taken together, the findings of fulminant liver failure despite maximum medical therapy, hypoglycemia, hyperuricemia, and coagulopathy made AFLP more likely. Furthermore, the patient's abdominal ultrasound findings were very unique in the findings of increased echogenicity which were consistent with steatohepatitis and help support a diagnosis of AFLP. These changes are not typically seen in ICP, preeclampsia, or HELLP syndrome.

As the patient had deteriorated rapidly, we started an emergent transplant evaluation. On hospital day 3, the patient's liver function tests continued to trend upward (peak AST 11,730 IU/L and peak ALT 4190 IU/L) and she developed new onset thrombocytopenia. She also developed mild cerebral edema due to an acute rise in ammonia levels. Her infectious work-up returned negative. As she had no absolute contraindications for transplant, she underwent an orthotopic transplant on hospital day 4.

**Figure 1:**
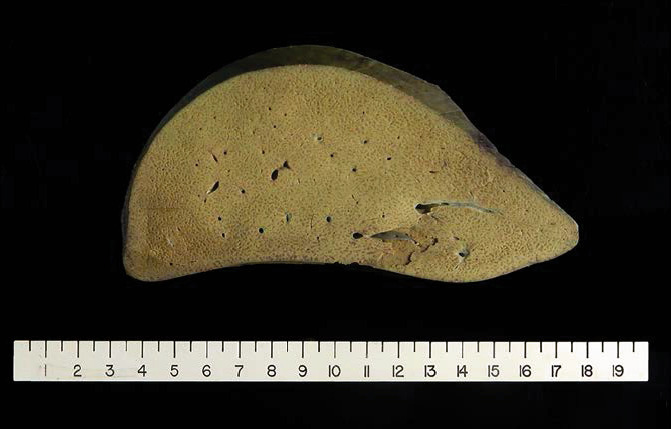
Gross examination of a portion of the explanted liver exhibiting pale-yellow discoloration indicative of steatosis.

**Figure 2:**
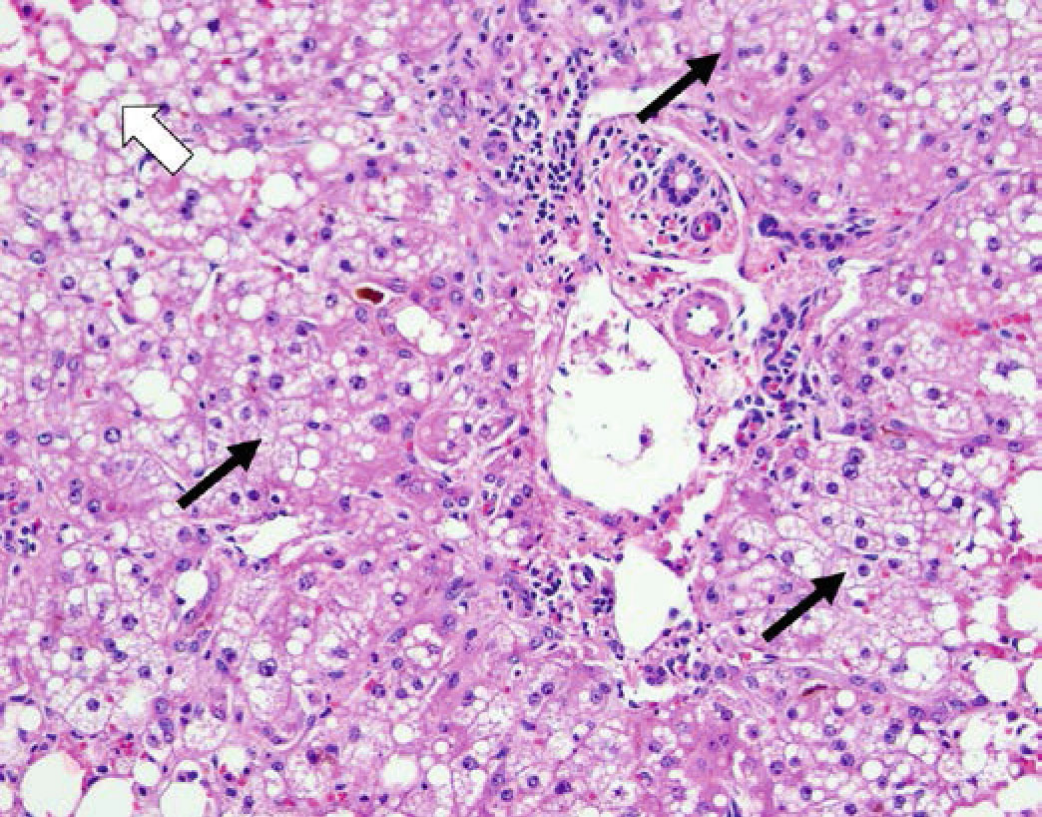
Histologic image from the liver explant demonstrating abundant microvesicular steatosis (black arrows) as well as macrovesicular steatosis (white arrow).

Unfortunately, her operative course was complicated by severe bleeding during the procedure and she eventually succumbed to the complications. Gross examination of her explanted liver showed a pale-yellow discoloration indicative of steatosis (Figure 1). Pathologic assessment of the native liver showed extensive mixed macrovesicular and microvesicular steatosis, normal bile ducts, and absent iron accumulation (Figure 2). This was most consistent with AFLP. The remainder of her laboratory studies returned negative.

## 3. Discussion

AFLP is a potentially fatal obstetric complication of pregnancy which is characterized by acute hepatic failure secondary to fatty infiltration of the liver. Subsequent complications of liver failure in AFLP include encephalopathy, renal insufficiency, hypoglycemia, coagulopathy, and respiratory failure. AFLP has an incidence ranging from 1 : 7000 to 1 : 15000 with most cases occurring in the third trimester [1]. Rarely, AFLP can occur in the second trimester, as seen in our patient. To date, there are only three reported cases of AFLP in the second trimester [3–5]. AFLP can also occur postpartum. In one study of 133 patients, only 9.8% of cases occurred postpartum [2].

To understand the pathophysiology of AFLP, the importance of the normal change of metabolism of fatty acids seen in pregnancy is crucial. During a normal pregnancy, there is a physiologic decrease in the oxidation of both long and medium chain fatty acids which subsequently lead to an increased maternal level of fatty acids during the course of the pregnancy, thus predisposing patients to the hepatotoxic effects of fatty acids [6]. Levels of free fatty acids increase particularly late in gestation which may explain why AFLP most commonly occurs in the third trimester. The hepatotoxicity caused by fatty acids causes a microvesicular fatty steatosis of the liver which impairs downstream production of coagulation factors, fibrinogen, and cholesterol and impairs the detoxifying function of the liver. There is also literature that suggests that these same fatty acids are also toxic to the pancreas with many cases of AFLP-associated pancreatitis being reported [7]. Furthermore, these increased fatty acids can predispose placental dysfunction which ultimately increases the risk of hypoxemic injury to the fetus [8].

More recently, our understanding of AFLP has improved after the close relation of fetal fatty acid oxidation disorders to maternal acute fatty liver disease. More specifically, the fetal deficiency of the enzyme long-chain 3-hydroxyacyl-coenzyme A dehydrogenase (LCHAD) which is a component of the mitochondrial beta-oxidation of fatty acids causes accumulation of long fatty acids [1, 6]. These same fatty acids can then cross over to the maternal circulation. This can then not only trigger the hepatotoxic effects to the maternal liver but also affect the mother's own mitochondrial beta-oxidation leading to further accumulation of free fatty acids. The incidence of liver disease can be as high as 75% in mothers who carry fetuses with LCHAD [6].

AFLP carries an autosomal recessive inheritance. Therefore, if genetically inherited, the mother is naturally at least a carrier of the mutated gene and therefore already at risk for the hepatotoxic effects of the fatty acids. Despite this known deficiency in the pathophysiology of AFLP, not all case reports have shown a fetal deficiency of this enzyme. Therefore, further pathophysiology pathways of this disease may exist.

Currently, there are no standardized guidelines on the approach to the diagnosis of AFLP. The Swansea criteria may help in making the diagnosis of AFLP when suspicion for the diagnosis is high (Table 1). The Swansea criteria has been prospectively validated for the diagnosis of AFLP whereby 6 or more clinical symptoms, laboratory, imaging, or pathology features are identified with the absence of another explanation [6]. In one study, the Swansea criteria had a sensitivity and specificity of 100% (95% CI 77% to 100%) and 57% (95% CI 20% to 88%), respectively, with a positive and negative predictive values of 85% and 100%, respectively [9]. Ultrasound findings of increased echogenicity may suggest steatohepatitis but is nondiagnostic on its own. A prospective national study in the United Kingdom by Knight et al. demonstrated that only 27% (*N* = 45) of patients with AFLP had abdominal ultrasound scan showing ascites or bright liver [10]. MRI has been suggested as a potential tool to detect fatty liver disease. In an observational study in France, five patients diagnosed with AFLP per the Swansea criteria had increased detectable fat on magnetic resonance imaging that had disappeared within 2 weeks postpar tum [11].

The mainstay of therapy is prompt delivery of the fetus. Otherwise, treatment is largely supportive with particular emphasis on treating complications of liver failure including hepatic encephalopathy, hypoglycemia, disseminated intravascular coagulation, acute renal failure, hepatic rupture, gastrointestinal bleeding, and acute respiratory distress syndrome. Patients often require admission to the intensive care unit for the management of the above [1]. Unfortunately, despite aggressive supportive care, fulminant liver failure can be inevitable and necessitating the need for liver transplantation as a last resort. Currently, there are no specific criteria for determining the need for liver transplantation in AFLP but case reports of AFLP warranting liver transplantation have generally been due to worsening clinical features such as encephalopathy, lactic acidosis, and worsening liver failure despite aggressive medical therapy [1].

According to a recent 2019 retrospective review of the national Scientific Registry of Transplant Recipients, data of all females in the United States undergoing liver transplantation due for AFLP (*N* = 18) from 1991 to 2015 had similar early survival outcomes (patient days from transplant until hospital discharge: median 21 days) compared to other groups (17 days and 13 days in the acetaminophen and “other ALF” groups, respectively, *P* = 0.002) [12]. Similarly, late survival outcomes were similar with cumulative 5-year patient survival outcomes being 73% (95% CI, 36-90) in the AFLP group compared to the acetaminophen group being 77% (95% CI, 70-83, *P* = 0.63) and “other ALF” being 82% (95% CI, 78-86, *P* = 0.67). However, the cumulative 5-year graft survival was significantly lower among AFLP patients (54%, 95% CI = 27-76) compared to liver failure secondary to acetaminophen (70%, 95% CI = 63-77) and “other acute liver failure” (76%, 95% CI = 72-80) groups.

The overall maternal mortality rates for AFLP with or without liver transplantation have markedly decreased from approximately 85% in the 1980s to approximately 10-15% in the 2000s [1]. This significant improvement is due to better recognition of AFLP with earlier diagnosis and, consequently, earlier delivery of the fetus and better obstetric intensive care. In a recent retrospective cohort study by Gao et al., amongst 133 patients with AFLP in China, the following risk factors were associated with adverse maternal outcome: male fetus, postpartum diagnosis of AFLP, intrauterine fetal death, disseminated intravascular coagulation, prolonged prothrombin time, and activated partial thromboplastin time [2]. There are currently no demonstrable patterns of recurrence of AFLP in women who have previously been affected with AFLP in their prior pregnancies; however, there are few case reports of recurrent episodes of AFLP [1, 13].

Our case highlights that AFLP can rarely occur earlier in the course of pregnancy and postpartum and should therefore remain in the differential for acute hepatic failure in a pregnant patient.

## Figures and Tables

**Table 1:** The Swansea criteria for the diagnosis of acute fatty liver disease of pregnancy. Six or more of the following features need to be present in the absence of another explanation to diagnose AFLP.

Symptoms	Vomiting
	Abdominal pain
	Polydipsia/polyuria
	Encephalopathy

Laboratory	Leukocytosis
	Elevated transaminases
	Elevated ammonia
	Elevated bilirubin
	Elevated urate
	Hypoglycemia
	Coagulopathy
	Renal impairment

Imaging	Ascites or bright liver on ultrasound

Pathology	Microvesicular steatosis on liver biopsy
